# Beyond enzymes and organic acids, solid-state fermentation as an alternative for valorizing fruits and vegetable wastes into novel bio-products in a circular economy: A critical review

**DOI:** 10.3934/microbiol.2025021

**Published:** 2025-06-24

**Authors:** Ramesh C. Ray, Sudhanshu S. Behera, Omojola Awogbemi, Balwinder Singh Sooch, Hrudayanath Thatoi, Subhashree Rath, Noé Aguilar-Rivera

**Affiliations:** 1 Center for Industrial Biotechnology Research, Siksha ‘O’ Anusandhan (SOA) Deemed to be University, Bhubaneswar, India; 2 Department of Biotechnology, National Institute of Technology, Raipur, India; 3 Department of Mechanical and Industrial Engineering Technology, University of Johannesburg, South Africa; 4 Enzyme Biotechnology Laboratory, Department of Biotechnology and Food Technology, Punjabi University, Patiala, India; 5 Universidad Veracruzana, Facultad de Ciencias Biológicas y Agropecuarias, Km. 1 Carretera Peñuela Amatlán de los Reyes S/N. C.P. 94945, Córdoba, Veracruz, México

**Keywords:** fruit and vegetable waste, solid-state fermentation, exopolysaccharides, biosurfactants, antimicrobial agents, carbon dots, lifecycle assessment

## Abstract

The magnitude of the global fruit and vegetable waste (FVW) generated and its contribution to environmental pollution and greenhouse gas emissions are alarming and necessitate appropriate remediation measures. In addition to typical FVW applications such as landfilling and manure production, our previous article critically explored the added value of FVWs for producing enzymes and organic acids by deploying various microbial processes. However, with the advancement of novel solid-state fermentation (SSF) technology, several products (other than enzymes and organic acids) have been developed from FVWs. This review article addresses the valorization of FVWs into the production of various bioproducts (i.e., microbial inoculants, single-cell proteins, aquafeeds, bioinsecticides, antimicrobial agents, or prebiotics), platform chemicals (i.e., polyphenols, biocolorants, exopolysaccharides, biosurfactants, biocomposites, or carbon dots), and biofuels. Upscaling and downstream aspects, techno-economic feasibility reports, and lifecycle assessments are also covered in the article. Rather than an overburden, FVWs can be regarded as a potential substrate for SSF, and successful transformation to novel bioproducts further contributes to a circular economy.

## 1. Introduction

A substantial amount of waste, estimated as 25%–30% of the total raw materials, is produced annually from the industrial processing of vegetables, fruits, roots, and tubers [1]. These wastes, primarily composed of seeds, skins, rinds, and pomace, are often discarded as by-products. Most of these unwanted materials are deposited in dumpsites and landfills and are recycled as animal feed or burned as an alternative [1]. Fruit and vegetable wastes (FVWs) typically comprise sugar, starch, proteins, phenolic phytochemicals, and minerals. They should not be regarded as waste but considered viable raw materials for several industrial production processes [2]–[6]. The sugar and starch in FVWs act as a source of carbon and protein, providing nitrogen, nutrients, and moisture and establishing ideal conditions for the growth of microorganisms. This opens enormous opportunities for their reutilization in processes of solid state fermentation (SSF) [7]. For example, for developing a variety of value-added commodities, FVWs can be utilized as solid support, carbon, and nutrients in SSF processes. In recent past, some reviews have dealt with the valorization of FVWs into bioproducts such as enzymes, organic acids [8], single-cell proteins, single-cell oils, and phenolic bioactive compounds, as well as their extraction procedure and possible applications as adsorbents, nanoparticles, bioethanol, and biogas. Even so, there is no comprehensive documentation of potential value-added products generated from FVWs using the SSF system [4],[8]–[15].

SSF refers to the phase during which microbial growth and product generation occur on the surface of solid materials. This process happens when there is no moisture or when moisture is completely absorbed into the solid matrix [16]–[19]. SSF presents lower capability when compared with submerged fermentation systems, but is capable of providing higher product yields, higher efficiency, lower energy requirements, and lower downstream processing; as such, it is of enormous importance in industrial operations [20]–[22]. Due to their availability, accessibility, and low cost, the reuse of FVWs in SSF processes is of particular interest, in addition to being an environmentally friendly alternative to their disposal. Before starting any fermentation phase, different factors such as microorganisms, FVWs used, water activity, temperature, aeration, and fermenter design should be considered for an effective SSF process. Single pure cultures, diverse recognizable cultures, or a consortium of mixed indigenous cultures or genetically modified microorganisms may be considered as the inoculum used in SSF. This review discusses the potential bioproducts (except microbial enzymes and organic acids) generated from FVWs using SSF technology.

The generation of enzymes, organic acids, and other materials from FVWs is excluded from this review since it has already been described [8],[23],[24]. Initially, an overview is provided regarding the production of such wastes, as well as their availability, properties, and biochemical composition. Then, a brief introduction of SSF is given, followed by a discussion of potential and novel applications of FVWs in SSF processes for obtaining value-added commodities, life cycle assessments, research gaps, and future challenges and perspectives.

## 2. Solid state fermentation (SSF)

The SSF system includes three major components: Substrates, microorganisms, and bioreactors.

### 2.1. Substrate

Sugarcane bagasse, cassava bagasse, cereal (rice, oat, and wheat) bran, coffee pulp and husks, and FVWs (fruit and vegetable peels and seeds) are the most suitable agroresidues used in SSF [23]. These materials, including FVWs, comprise cellulose, lignin, hemicellulose, starch, pectin, ash, and dietary fibers [20],[21],[25]. These agroresidues usually act as solid support for absorbing nutrients and biomass growth and as a source of carbon and nutrients [19],[23]. Nutrient supplementation (phosphorus, sulfur, potassium, magnesium, calcium, iron, zinc, manganese, copper, cobalt, etc.) is often required to achieve maximum microorganism growth and product yield [21],[23]. Key factors, such as cost and availability, are considered when selecting a residue as the substrate or support in SSF [26]. Nonetheless, significant factors such as crystallinity, permeability, surface area, and particle size must be considered for the SSF process [23].

### 2.2. Microorganisms

Regarding SSF, the selection of microorganisms and substrates are the two most significant factors. Filamentous fungi are appropriate for the SSF process as they replicate their natural habitats, allowing them to produce diverse metabolites such as enzymes and organic acids [23]. Yeasts are also well-suited for SSF because they can flourish in low water-activity environments. Certain bacterial species, like *Bacillus subtilis*, *B*. *megaterium*, *B. thuringiensis*, and *Lactobacillus* spp., have been documented to effectively produce enzymes and other metabolites in SSF [20]. Additionally, actinomycetes, specifically *Streptomyces* spp., are also known to be conducive to SSF, as they possess traits including abundant colonization of solid residue, the synthesis of various degrading enzymes including cellulase and β-glucosidase, and high tolerance to extreme environmental situations [23].

### 2.3. Bioreactors

Based on the mixing technique employed, bioreactors in SSF are divided into two categories: Stirred bioreactors (horizontal drum or stirred drum) and static bioreactors (fixed bed, perforated trays). Additional classifications are also based on the type of aeration used (with or without forced aeration) or the mixing method used [20],[27]. These include different configurations of tray reactors, packed-bed reactors, horizontal drum reactors, and fluidized bed bioreactors, each with its merits and drawbacks. This shows the importance of developing improved or innovative bioreactors [23]. Bioreactor details are not covered in this review.

## 3. Fruit and vegetable wastes overview: Availability and properties

After processing, the inedible parts of fruits and vegetables, such as seeds, leaves, peels, pods, nuts, and skins, make up approximately 10%–50% of the total weight of the fresh produce (Table 1) [3],[28]. This waste leads to disposal challenges and could potentially result in severe pollution issues, as well as the loss of valuable biomass and nutrients [3]. Fruit processing and conversion industries alone generate over 0.5 billion tons of waste globally [29]. To determine how these waste products can be used as raw materials and to propose appropriate methods for turning them into value-added products, it is essential to characterize FVWs as a distinct category of solid waste (biomass) [30]. The composition of the waste also impacts the total yield and kinetics of the biological reaction during the digestion process. Waste characterization and analysis can be conducted in physical, chemical, and biological terms [30]. Physical characterization of FVWs involves measurements of volume, weight, carbon, nitrogen, ash, moisture, total solids, volatile solids, color, odor, temperature, and pH, among others [6]. Chemical studies include measurements of cellulose, hemicellulose, lignin, total organic carbon, total nitrogen or protein, phosphorus, sulfur, starch, sugar, and toxic compounds, among others [30],[31]. Biological characterization involves identifying the presence of pathogens, mycotoxins, toxic metabolites such as the cyanogenic glucosides linamarin and lotaustralin in cassava bagasse, and antinutrient factors [28],[32],[33]. Additionally, for FVWs in semi-solid or semi-liquid states, it is important to measure biological oxygen demand (BOD) and chemical oxygen demand (COD). Most FVWs are also rich sources of nutrients such as vitamins, minerals, dietary fibers, and bioactive peptides [4],[8],[34]. Table 2 provides some physical and chemical characteristics of selected FVWs.

**Table 1.** Percentage of waste generated from fruit and vegetables.

Fruits/vegetables	Waste nature	Quantity of waste (%)	Reference
Apple	Peel, pomace, seed	15–25	[28]
Mango	Peel, stone	20–45	[28]
Citrus	Peel, rag, seed	50	[28],[35]
Tomato	Skin, core, seed	20	[28]
Pineapple	Skin, core	33	[28]
Grape	Stem, skin, seed	20	[28]
Guava	Peel, core, seed	10	[28]
Blueberry	Stem, skin, seed	30	[36]
Coconut	Husk	30	[36]
Date palm	Stem, skin, seed	30	[36]
Kiwi	Stem, skin, seed	30	[36]
Olive	Stem, skin, seed	30	[37]
Durian	Stem, skin, seed	60	[36]
Banana	Peel	35	[28]
Potato	Peel	15	[28]
Onion	Outer leaves	10	[28]
Pea	Shell	40	[28],[38]
Cassava	Peel, bagasse	15–20	[39]
Sweet potato	Peel, bagasse	10–15	[40]

**Table 2.** Biochemical compositions of FVWs.

Fruit-industrial waste	Biochemical composition (% w/w)								
	Cellulose	Hemi-cellulose	Lignin	Ash	Total solids	Moisture	Total carbon	Total nitrogen	References
Potato peel waste	2.2	–	–	7.7	–	9.89	1.3	0.48–0.8	[31],[41]
Potato mesh	17–25	10-15	–	6–12	1.7–19.0	85–87	–	0.48–0.8	[31]
Cauliflower waste	17.32	9.12	5.94	4.32–5.76	–	81–89	34.48	13.8	[42]
Pea pod waste	32.08	21.12	21.58	4.8–5.20	11.0–39.0	73.5–88.5	–	10.58	[43]
Onion (onion tops peelings and whole bulbs)	–	–	–	4.7–4.8	91.0	82.0–92.6	–	–	[31]
Tomato wastes	30–32	5-18	–	3.1–5.3	7.0–22.4	85–90	–	2.72–3.52	[31]
Carrot peels	13–52	12–19	–	3.8–8.9	7.0–11.0	–	–	0.8–1,28	[31]
Sugar beet (pulp, silage, and leaves)	26.3	18.5	2,5	4.8	87.5	12.5	–	–	[31],[44]
Orange peel	9.21%	10.5%	0.84%	3.5%	–	11.86	–	–	[44]
Coffee skin	23.77	16.68	28.58	5.36	–	–	C/N 14.41		[44]
Apple pomace	5–10	4–25	15–25	5.8–6.7	–	15–28	–		[6]
Pineapple peel	35–50	19.7–35	5–10	4.6–5.8	93.6	75–80	40.8	0.99	[45]
Banana peel	12.17	10.19	16.0	5.01	–	9.65	40.24	1.38	[15],[41]
Mango peel	9.2	14.5	4.25	–	–	–	–	–	[36]
Coconut husk	34	21	27	–	–	–	–	–	[36]
Papaya peels	–	–	–	3.15–5.25	31–45	54–68	38.10	1.49	[44]
Pomegranate peels	–	–	–	–	–	–	–	–	[46]
Rambutan peel	24.28	11.62	35.39	–	–	–	–	–	[36]
Almond shell	32	28	−32	–	–	–	–	–	[47]
Durian	69.4	13.1	15.45	–	–	–	–	–	[36]
Palm oil empty fruit bunch	37.26	14.62	31.68	–	–	–	–	–	[36]

Further, the chemical compositions of waste biomass affect bioproduct generation. To cite an example, a case study on the composition of false banana (*Ensete ventricosum* [Welw.]), a staple food in Ethiopia, is described. The plant produces huge quantities of biomass residues, mainly from the pseudostem (fiber bundles collected after scraping the leaf sheaths to produce starchy food) and the inflorescence stalk [48]. The results suggest different potential uses for these products. The fiber bundles could be utilized as a feasible source of fiber and feedstock for paper pulp processing, possibly after removing hemicellulose. Meanwhile, the inflorescence stalk has nutritional content for food and fodder and can also be utilized for sugar fermentation products such as bioethanol and biobutanol.

## 4. Bioproducts derived from FVWs in SSF

**Figure 1.**
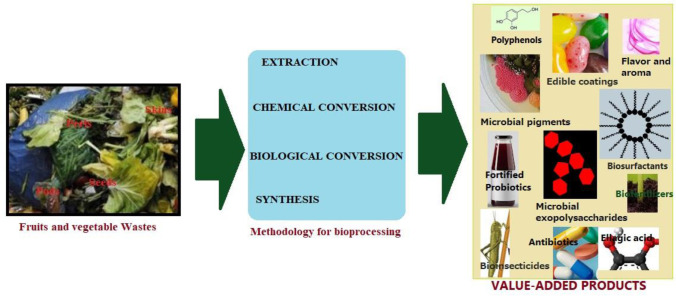
Biovalorization of fruits and vegetable waste into some important value-added products.

SSF can use a wide variety of FVWs as substrates; thus, it is an excellent candidate in the framework of the circular bioeconomy to change the status of waste from feedstock to high-value-added products. The development of SSF was boosted in the previous decade by scientific efforts devoted to producing hydrolytic enzymes and organic acids [4]. Nowadays, SSF has expanded to other valuable products: Immobilization carriers, bioactive compounds, polyphenols, biosurfactants, biopesticides, aromas, pigments, and single-cell proteins (SCP), among others (Figure 1). This section explores the conditions to obtain the main emerging SSF products from FVWs and briefly highlights and discusses the challenges related to the scale-up and down-streaming of these processes.

### 4.1. FVWs as immobilization carrier

The usage of fungal and bacterial immobilization carriers in SSF has been widespread as feedstock for low-cost and sustainable generation of enzymes, organic acids, antioxidants, pigments, growth regulators, and nutraceuticals for the agricultural, food, and pharmaceutical industries, and fermentation, environmental detection, and clinical diagnosis applications. Using FVWs as solid support offers several benefits [49]. First, it significantly enhances product stability in harsh environments. Second, it lowers production costs due to the low-cost, biodegradable, and less toxic nature of FVWs. Third, it provides convenience, lessens the risk of contamination of the products, and provides feed for microorganisms [27],[49]. The adsorption mechanisms of the FVWs involved can be physical, chemical, or a combination of both; the impact on productivity depends on various factors such as porosity, surface charge, and functional groups [49]. Physical adsorption is primarily based on weak intermolecular forces like van der Waals forces between the bioproducts (i.e., enzymes, pigments) and the FVW surface. It is generally reversible and can be influenced by factors like temperature and pH. On the other hand, chemical adsorption involves stronger bonds, such as covalent bonds, between the bioproducts and the carrier (i.e., FVWs). It is often irreversible and can result in a more stable immobilization [50].

An evaluation of various FVWs, including lime peel, orange peel, apple pomace, pistachio shell, wheat bran, and coconut husk, was conducted to determine their suitability as carriers in SSF [50]. These materials were analyzed based on their physical-chemical properties (water absorption index and critical humidity point) and microbiological characteristics (growth of *Aspergillus niger* Aa 20). The study found that coconut husk, apple pomace, lemon, and orange peels showed high potential as immobilization carriers in SSF due to their impressive water absorption capacity. Other substrates used as immobilization carriers included French beans (*Lablab purpureus*), black-eyed peas (*Vigna unguiculata*), cassava and sweet potato bagasse, and apple and grape pomace, as well as other forms of vegetable and fruit peels [7],[19],[37],[38],[43],[51].

### 4.2. Single-cell proteins and aqua feed

Single-cell proteins (SCP) are obtained from appropriate microorganisms like bacteria, fungi, yeast, and algae cultivated on carbon sources for synthesis [52]. SCP can be used as a protein supplementation for humans and livestock, substituting expensive traditional protein sources, including soy meal and fish meals, to address protein scarcity/deficiency [4],[9],[28],[52],[53].

One potential solution to the low protein content in FVWs is the utilization of food-grade microorganisms, primarily fungi and yeasts, to convert them into biomass with higher nutritional values, particularly regarding protein and vitamin contents, demonstrating improved digestibility [4],[54]. The raw FVWs, fats, soluble sugars, vitamins, and amino acids are upgraded by SSF and are deployed as raw materials for animal feed production [55],[56]. Using SCP from FVWs is expected to address the global deficiency of protein-rich foods and animal feed and solve waste disposal and associated pollution problems [1]. For example, sweet potato bagasse has been enriched with amylolytic yeast and mycelial fungi for protein enrichment in SSF [57],[58]. Similarly, apple pomace was utilized for SSF using a combination of different microorganisms, significantly increasing protein and mineral contents [59],[60].

SCP has been generated by SSF from various agricultural wastes, such as pomegranate, orange, banana peels, grape pomace, and watermelon wastes, using microorganisms such as *Saccharomyces cerevisiae* [61]–[63]. The mixture of potato peels and ammonium chloride was also utilized for the production of SCP using the food-grade fungus *Pleurotus ostreatus* [64]. Other examples include the production of SCP from sources such as Jerusalem artichoke extracts using a marine yeast, *Cryptococcus aureus* G7a, and from FVWs using *S. cerevisiae* in SSF, which showed significant protein production from cucumber peels compared to orange peels [65],[66]. Mango peels and seed meal were also fermented for SCP production in SSF using different yeasts and lactic acid bacteria, resulting in notable nutritional changes [67].

Recently, scientists have investigated SCP production from apple, orange, tomato, and pea peels by growing *S. cerevisiae* in SSF [52]. Moreover, FVWs are used in aquaculture as feed products, supplemental feed, or pond fertilizers in some tropical regions of Asia and Africa [68]–[70].

The nutritional value of palm kernel meal pretreated with *Trichoderma koningii* in SSF as a dietary constituent for red hybrid tilapia (*Oreochromis* sp.) has been studied [71]. Furthermore, investigations on the nutritional status of SSF products from a blend of vegetable waste powder and a variety of oil cake mixtures resulted in major improvements in crude protein and amino acids [70]. This represents a summary of some key findings in SCP production from various agricultural and food wastes, as well as SCP's potential benefits and applications in addressing protein scarcity and waste management.

### 4.3. Microbial inoculants

Arbuscular mycorrhiza (AM) fungi are rich biofertilizers commonly used for fruits and plantation crops. AM fungi are obligate symbionts and cannot be multiplied in synthetic media. The only production method is immobilizing carrier materials like FVW, an excellent example of SSF [72]. The efficacy of *Trichoderma*, *Penicillium*, and *Rhizopus* spp. for solid-state production was investigated on apple and cranberry pomace [73]. The best growth rate of these fungi was achieved with apple pomace assisted with CaCO_3_, water, and fish protein hydrolysates. Apple pomace was a more suitable substrate than cranberry pomace for cultivating *Trichoderma* spp. and *Penicillium* spp., but a comparatively inferior substrate for breeding *Rhizopus oligosporus* [74].

Further, *Trichoderma* inoculants cultivated on apple pomace showed improved performance in peas cultivated in potting soil [73],[74]. The study implied the suitability of apple and cranberry pomace as immobilizer carriers of fungal inoculants. It was reported that sterilized cassava roots are randomly deployed to multiply *Bradyrhizobium japonicum* in Thailand [75].

### 4.4. Food-grade fungi/mushrooms and fortified probiotics

The cultivation, growth, and performance of *Lentinus edodes* (also known as Shiitake mushroom) were evaluated on cassava bagasse [76], with similar results as reported when *Pleurotus sajor-caju*, another edible mushroom, was cultivated on banana and rice straw [77]. Apple, strawberry, cranberry pomace, banana leaves, and potato wastes also proved to be suitable for growing *L. edodes*, *Volvariella volvacea*, and *Pleurotus* sp. [73] (Table 3). Banana stalks also produced *Pleurotus sajor-cajun* [78]. Residues from Pequi (*Calophyllum brasiliense*) and guava (*Psidium guajava*) fruits were effectively employed as substrates for *Pleurotus sajor-cajun* production during an SSF. The protein-rich wastes are used as low-cost livestock feeds [79]. Banana leaves could grow *Volvariella volvacea*, an edible mushroom. Various FVWs were used as a substrate for the effective cultivation of some edible mushrooms [80]. The authors reported that bottle gourd peel, carrot peel, drumstick peel, and papaya peel in the media boost the growth of *P*. *sajor-caju*, *Lentinus tuberregium*, and *Calocybe indica*.

Probiotics are “live microorganisms that, when consumed in an adequate amount, confer a health benefit on the host” [81]. Lactic acid bacteria are usually utilized as probiotics to prepare different fermented functional food items such as yogurt, curd, or acidophilus milk [82]. Some of the most frequently used probiotic microorganisms in fermented functional foods are *Lacticaseibacillus rhamnosus*, *Limosilactobacillus reuteri*, *Lactiplantibacillus plantarum*, *Lacticaseibacillus casei*, *Lactobacillus acidophilus*, bifidobacteria, *Bacillus coagulans*, *Escherichia coli* strain Nissle 1917, certain enterococci, especially *Enterococcus faecium* SF68, and the yeast *Saccharomyces boulardii* [82]–[84]. In semi-solid fermentation, these probiotic fermented foods are often enriched with fruits and vegetable peel powder to enhance properties such as dietary fiber, antioxidant, and oligosaccharide content. Further, FVW powder acts as a prebiotic to support the growth and activity of naturally present or added probiotics. Probiotic yogurt produced from a mixture of apple, banana, and passion fruit peel showed better rheological properties and populations of lactobacilli and bifidobacteria [85]. To develop fat and sugar-free probiotic yogurt, a composite fruit peel powder derived from orange, passion fruit, and pineapple was used in varying proportions (0.5%–1.0%, w/v) [86]. The firmness and consumers' acceptability increased, and high lactic acid bacteria counts were observed in yogurt containing 0.5% peel mixtures. Mango and pineapple peel powder were added to milk in kefir (a type of fermented milk consumed in Eastern Europe, Russia, and the North Caucasus, and prepared with kefir grains) to enhance antioxidant and rheological properties [87],[88].

**Table 3.** Growth and cultivation of various edible fungi and probiotic microorganisms on FVWs.

Fungi/probiotics	FVW	References
*Lentinus edodes*	Cassava bagasse, apple, strawberry, cranberry pomace, banana leaves, and potato wastes	[73],[76]
*Pleurotus sajor-caju*	Banana stalk and rice straw, Pequi, guava	[77]–[79]
*Volvariella volvaceae and Pleurotus* sp	Apple, strawberries, cranberry pomace, banana leaves, and potato wastes	[73],[80]
Lactobacilli and bififobacteria	Apple, banana, and passion fruit peel	[85]
Kefir grains	Mango and pineapple peel powder	[87],[88]

### 4.5. Bioactive compounds

Bioactive compounds are additional dietary components employed as feedstocks in the food processing, cosmetics, and body care industries. Popular bioactive compounds comprise secondary metabolites like dietary fibers, essential oils, phenolic phytochemicals, alkaloids, plant growth regulators, antibiotics, and food-grade pigments [89]. In recent studies, targeted investigations into the use of SSF for the production of bioactive compounds have escalated [6],[10],[90].

FVWs rich in soluble and insoluble fibers are employed by lignocellulolytic fungi in SSF because they possess enzymes such as ligninases, cellulases, peroxidases, polyphenol oxidases, and hemicellulases that can break down complex fiber structures. Some phenolic compounds are discharged during the hydrolysis of lignin. Fungi also synthesize compounds with profound health benefits, such as antitumoral, antimicrobial, antioxidant, and antiviral activities, including mycophenolic acid, phenylacetates, anthraquinones, benzofurans, and alkenyl phenols [7],[10],[19],[91]. Additionally, fungi produce polysaccharides with important health-promoting properties [92]. Extracting biomolecules (antioxidants, dietary fibers, proteins, etc.) from FVWs by non-thermal processes could efficiently produce highly purified functional ingredients.

The bioactive compounds available in waste from fruits such as apples, avocados, bananas, citrus, grapes, mangoes, plums, peaches, and apricots, and vegetables such as beetroot, carrot, and cauliflower have been characterized. Their continued utilization in the food, environmental, cosmetic, and pharmaceutical industries has been extensively discussed [6]. Furthermore, the extraction of these bioactive compounds from FVWs using different methods such as Soxhlet extraction, hydro-distillation, and maceration [10] as well as purification and valorization at industrial levels has been reviewed [10],[93]–[95].

### 4.6. Polyphenols, ellagic acid, and edible coatings

Polyphenols are a group of compounds found naturally in fruits, vegetables, plant-based foods, and beverages. They are used to neutralize toxic free radicals and prevent cell damage. Polyphenols prevent inflammation and help minimize the risk of cancer, heart-related ailments, diabetes, and other chronic health challenges. Notable examples include flavonoids, phenolic acids, lignans, stilbenes, and tannins [89]. FVWs are also rich in soluble and insoluble fibers and are often used by lignocellulolytic fungi in SSF because they possess enzymes such as ligninases, cellulases, peroxidases, polyphenol oxidases, and hemicellulases that can break down complex fiber structures. Some phenolic compounds are produced during the hydrolysis of lignin. Fungi also synthesize compounds with health advantages such as antitumoral, antimicrobial, antioxidant, and antiviral activities, including mycophenolic acid, phenylacetates, anthraquinones, benzofurans, and alkenyl phenols [7],[10],[19],[91]. Additionally, fungi produce polysaccharides with important health-promoting properties [92]. Extracting biomolecules (antioxidants, dietary fibers, proteins, natural colorants, aroma compounds, etc.) from FVWs by non-thermal processes could efficiently produce highly purified functional ingredients.

Ellagic acid is a natural polyphenol compound with antioxidant and anticancer properties. It is found in many fruits and vegetables, including berries, pomegranate, and nuts. Pomegranate wastes were processed using *Aspergillus niger* and *S. cerevisiae* in SSF. Ellagic acid recovery yields were evaluated using ultrasound and microwave-assisted extraction with a 7:3 water/ethanol ratio [96]. Surprisingly, the outputs obtained from *S. cerevisiae* fermentation were found to be five times higher than those obtained from *A. niger*.

The SSF of fruit wastes produces phenolic antioxidants, which can be included in foods using coatings/wax-based films to prevent alterations in their quality [97]. Banana peels are used in edible antimicrobial coating for food packaging [98],[99].

### 4.7. Flavor and aroma compounds

As defined in the European Community guidelines 88/388/EWG and 9/71/EWG, the first category of the aroma classification specifies the regulation for food to be labeled with “natural flavor” [100]. Natural flavors can be classified as chemical compounds with aromatic properties derived from plants, animals, or microorganisms and processed by physical, enzymatic, or biological means. Typically, the extraction of that type of compound is accomplished by chemical synthesis or extraction from natural products; however, new cost-cutting methods such as SSF have been introduced due to the economic consideration of these processes [24],[101]. In food flavoring, microorganisms are essential in producing natural compounds [100],[102]–[104]. SSF has generated aromas such as vanillin, 2-phenethyl alcohol, coconut-like flavor, fruit-like flavor, or nutty and roasted flavor [102],[105]–[107]. Some of the essential products developed from FVWs in SSF are shown in Table 4.

**Table 4.** Some essential products developed from FVWs in solid state fermentation (SSF).

Compounds produced	Nature of fruit/vegetable waste	Technology adapted	Scope of application	Reference
Immobilization carrier	Coconut husk, cassava, and sweet potato bagasse, vegetable and fruit peels	Grinding and thermal processing	Immobilizing agent	[7],[38],[50]
Bioioculant (*Trichoderma* spp.)	Apple pomace	SSF	Biofertilizer	[74]
Mushroom (*Pleurotus sajor*-*cajun*)	Banana stalks	SSF	Edible mushroom	[108]
Single-cell protein	Sweet potato bagasse/potato peels	SSF using food-grade fungi and yeast	Feed	[28],[57],[64]
Fish feed	Waste biomass from sweet potato distillery	Protein enrichment by fermentation with *S. cerevisiae*	Fish feed	[109]
Polyphenols	Grape seeds	Pulsed electric field	Antioxidants	[110]
Ascorbic acid	Lemon and orange peel	Aqueous extract	Vitamin C	[111]
Ellagic acid	Pomegranate waste	SSF (using *A. niger* and S. *cerevisiae*) followed by ultrasound and microwave-assisted 7:3 water/ethanol extraction	Antioxidants	[96]
Aromatic esters	Coffee husk, palm bran, and cassava bagasse	SSF	Flavoring agent	[112],[113]
Carotenoid	Apple pomace, citrus wastes, cabbage, watermelon husk, tomato peel, and peach peels	SSF	Colorant and antioxidant	[114]–[117]
Anthocyanins	Grape pomace	Supercritical solvent extraction	Colorant	[79]
Non-toxic red pigment	Coconut husk	Microbial processing by *Monascus* sp.	Colorant	[118]
Fructooligosaccharides	Date fruit byproducts	Saccharification and fermentation	Alternative sweetener	[119]
Xylitol	Banana peels	Microbial processing by *Candida tropicalis*	Low-calorie sweetener	[120]
Xanthan gum	Potato peels	Microbial processing by *Xanthomonas* spp.	Used in the food industry	[121]
Pullulan	Cassava bagasse	SSF using *Aureobasidium pullulans*	Food additive	[122]
Lovastatin	Orange peel	SSF with *Penicillium funiculus*	Antibiotic	[123]
Indole 3-acetic acid	Cassava bagasse	SSF with *Bacillus subtilis*	Plant growth regulator	[124]
Lycopene and carotenoids	Tomato pomace	Pulsed electric field	Colorant	[125]
Polyhydroxy butyrate	Orange peel waste	Microbial processing using the modified strain of *Bacillus subtilis*	Bioplastic	[126]
Biosurfactants	Orange peel, banana peel, potato peel, cassava bagasse, moringa residue	*Pseudomonas aeruginosa* PB3A, *Halobacteriaceae archaeon*, *Bacillus pumilis*, *Bacillus licheniformis*, *Rhodococcus*	Agricultural, biomedical, cosmetics, and pharmaceutical applications	[127]–[130]
Bioadsorbents	Pineapple, watermelon, pomegranate	Physical processing	Removal of toxic materials, heavy metals, and dyes	[14],[38]
Carbon quantum dots	Lemon peels, grapefruit peel, orange peels	Hydrothermal process	Nanoplatforms for biosensing, bioimaging, drug delivery, etc.	[131]–[133]
Bioethanol	Banana peel, carob pod, banana pseudostem	Microbial processing using *A. niger* and *S. cerevisiae*	Biofuel	[134]–[136]
Biogas	Papaya peels	Anaerobic digestion	Biofuel	[137]

According to the strain and culture specifications, fungi from the genus *Ceratocystis* develop an array of fruit-like or flower-like flavors [105],[138],[139]. Among various *Ceratocystis* species, *C. fimbriata* is reported to possess an impressive ability to synthesize esters. Production of fruit-based fragrance by *C. fimbriata* using various FVWs, such as apple pomace (combined with soybean or cassava bagasse), amaranth, and soybean, has been documented in solid cultures. A compelling pineapple flavor is perceived and recorded when this fungus is grown on SSF using coffee husk as a substrate. In contrast, a strong banana aroma is caused by adding leucine or valine [112]. Increased production of fruity aroma materials has been described using citrus pulp derived from fruit processing industries grown with *C. fimbriata* by SSF blended with soy bran and sugarcane molasses [140].

The deployment of various strains of yeast facilitates the creation of the aromatic compounds required. Apple pomace inoculated with *S. cerevisiae*, *Hanseniaspora valbyensis*, and *Hanseniaspora uvarum* was used to produce fatty acids and their resultant ethyl esters. Similarly, an inoculated substrate with *Hanseniaspora* sp. generated acetic esters and numerous volatile compounds of diverse chemical extraction [141]. The development of diverse aroma compounds by SSF using *Kluyveromyces marxianus* cultivated on cassava bagasse, giant palm bran, apple pomace, and other FVWs was recorded in an earlier study. This strain developed a fruity aroma of alcohols, esters, and aldehydes derived from feedstocks such as palm bran and cassava bagasse [113].

### 4.8. Microbial pigments

Nowadays, microbial colorants like carotenoids, melanins, and violacein, among others, are generated by adopting bioprocessing technologies from microorganisms [142]. For instance, *Blakeslee transport*, *Xanthophyllomyces dendrorhous*, *Penicillium oxalicum*, and *Ashbya gossypii* are used for the production of β-carotene, astaxanthin, and riboflavin, respectively [142]. Several authors have reported carotenoid production by fungi. *Blakeslea trispora* and *Phycomyces blakesleeanus* have been investigated for their possibility of carotenoid synthesis on small, pilot, and industrial scales [143].

β-carotene is an orange-red pigment found in fruits and orange-fleshed vegetables like carrots and sweet potatoes [144]. It is an antioxidant that converts to vitamin A and plays an important role in human health improvement. It reduces the risks of cardiac arrest, heart failure, and other macular degeneration conditions. Various bodily processes require essential nutrients, such as vision, cell differentiation, glycoprotein production, and bone formation, growth, and development. Their uptake is therefore crucial for the regeneration and improvement of numerous bodily systems [11],[145]. It has been reported that apple pomace is a good support for the formation of carotenoids using *Rhodotorula* sp. [114]. Further, co-culturing *Rhodotorula glutinis* and *Debaryomyces castelli* in SSF enhances biomass and carotenoid production (Table 3).

HPLC, FTIR, LC–MS, and MS are used for different analytical purposes in biochemistry and related fields. HPLC separates and identifies compounds based on their interactions with a stationary phase. FTIR analyzes the vibrational frequencies of molecules to identify functional groups. LC–MS combines the separation power of HPLC with the mass analysis capabilities of MS for detailed compound identification and quantification. MS measures the mass-to-charge ratio of ions to determine the molecular mass of compounds. Dynamic light scattering (DLS) is used to determine the size and size distribution of particles and molecules in solution, primarily in the submicron range. It measures the Brownian motion of particles, which is related to their size and diffusion coefficient. These techniques are employed in analyzing color pigments.

In SSF, using the fungus *Blakeslea transport*, MTCC 884 was studied to generate β-carotene from FVW (orange, carrot, and papaya peels). It was found that the formation of β-carotene was substantially enhanced by optimizing all parameters to achieve maximum yield. Various techniques such as HPLC, LC–MS, FTIR, and mass spectroscopy were used to characterize the extracted color. Mass spectroscopy of extracted color revealed the availability of β-carotene in high concentration. In addition, the estimated β-carotene was over 76%, indicating that these FVWs can be used for β-carotene purity development with excellent antioxidant properties [146]. β-carotene production by citrus wastes, cabbage, watermelon waste, tomato peel, and peach peels using *Blakeslea transport* ATCC 14271 and *B. transport* ATCC 14273 are some other interesting studies [115]–[117],[147]. Anthocyanin is another fruit dye that can be found in red grape pomace and similar species of grapes. It is a natural phenolic compound noted for its variety of colors and pigmentations [145]. Its health benefits include minimizing oxidative stress, free radical scavenging, reducing cancer and disease risk, and regulating cholesterol [148]–[150]. Laboratory productivity of 450 mg of anthocyanin per 100 g of pomace dry weight (dw) has been achieved [79].

The potent production of nontoxic red pigments has been attributed to *Monascus* species, particularly *M. purpureus* and *M. ruber*, which are commonly utilized as food additives for flavor modification, food preservation, and as colorants [4]. It has been reported that *Monascus* pigments are easily produced via SSF techniques. Various FVWs, such as pineapple waste, coconut husk, jackfruit seeds, and orange peel residue, are employed in SSF using *Monascus* spp. [118],[151]–[153]. Further, *M. purpureus* was more effective and preferable than *Penicillium purpurogenum* for the cost-effective production of pigments during SSF, yielding 9 absorbance units (AU) per gram of the dry fermented substrate [153]. One investigation on the appropriateness of cassava starch and bagasse as sources of carbon for pigment production and growth of *M. purpureus* has been conducted [154]. In contrast, some researchers have identified potential issues with the extensive production of pigments through SSF, such as inadequate production of unrefined pigments, increased labor costs, and more complex control challenges in comparison to submerged fermentation (SmF) [155],[156]. The applications of microbial pigments as food quality-enhancing agents have been reviewed by various researchers [11],[157],[158].

### 4.9. Microbial exopolysaccharides (EPSs)

Microbial EPSs are synthesized by microorganisms and secreted out of the cell. They have several applications in food, pharmaceuticals, cosmetics, brewing, and electronics [159]. They can function as thickening agents and facilitate the formation of gels, and in other food industry processes due to their colloidal fingerprint. The microbial EPSs can be generated in SmF and SSF.

Xanthan gum is the most commonly used microbial EPS produced by *Xanthomonas* spp. Xanthan is deployed as a food additive. The xanthan production in SSF was studied using various FVWs and different *Xanthomonas* spp. [121]. The solid residue generated from Jerusalem artichoke tubers and carob pods was used as feedstock for EPS production of pullulan by the yeast-like fungus *Aureobasidium pullulans* [160]. Similarly, cassava bagasse (residues left after the extraction of starch) and flour were employed in SSF for the production of pullulan and scleroglucan [122],[161]. Chitosan production by the fungus through SSF on sweet potato biomass was studied; chitosan yield was impressive and witnessed higher fungal growth as time increased [162] (Table 3).

### 4.10. Biosurfactants

Biosurfactants are surface-active compounds synthesized by microorganisms. They possess higher multifunctionality and biodegradability and lower toxicity than synthetic surfactants [163]. They have several applications in specific areas such as environmental, nutritional, household, agricultural, biomedical, cosmetics, and pharmaceutical industries. Fungi, yeast, and bacteria can produce biosurfactants during their cultivation. SSF is emerging as one of the most potent strategies for the production of biosurfactants, especially in order to overcome the problem of foam production encountered in submerged fermentation [24],[164]–[166]. Although there have been numerous investigations on biosurfactant production using agro-wastes in SSF, significantly less information is available on using FVWs as substrate [166].

A bacterial strain, i.e., *Pseudomonas aeruginosa* PB3A, was effectively isolated from samples containing oil-polluted materials. The strain was used for the extraction of biosurfactants using agricultural waste [167]. FVWs such as orange peel, banana peel, potato peel, cassava bagasse, and moringa residue were used for the production of biosurfactants in SSF using microorganisms such as *Bacillus pumilis*, *B. licheniformis*, *Halobacteriaceae archaeon*, *P. aeruginosa*, and *Rhodococcus* [127]–[130]. In another work on surfactant production by SSF, *B. subtilis* SPB1 was cultivated on a blend of olive leaf residue and olive cake flour. 30.67 mg of surfactant/g substrate (w/w dry weight) was produced [168]. *Halobacteriaceae archaeon* AS65 was able to produce biosurfactants using banana peel as an innovative substrate [169]. However, it is essential to critically track heat production in the SSF system, since differences in temperature will determine the generated surfactin homologs. Therefore, much optimization is required to make this approach achievable and affordable [130],[170].

### 4.11. Fructooligosaccharides, xylitol, and other low-calorie sweeteners

Fructooligosaccharides (FOSs), also known as oligofructoses, are oligosaccharides that, when ingested, provide significant health benefits (although they may cause digestive problems for certain persons). They can be applied as an artificial sweetener or dietary fiber due to their low caloric value. FOSs have essential functional components and properties because they can serve as a substrate for microflora in the large intestine, improving the gastrointestinal tract's overall health. Conventionally, FOSs are produced from sucrose and enzymes such as β-fructofuranosidase and fucosyltransferase, generated mainly by fungi such as *Aspergillus* spp., *Rhizopus* spp., *S. cerevisiae*, and *Lactobacillus acidophilus*, among others, to catalyze the bioconversion of sucrose into FOS. Synthesis of FOS is a two-step process in which an enzyme is produced and then used in a biotransformation process to generate FOS [171].

Among the FVW that have been effectively deployed for FOS production and FOS-producing enzymes (FFase) are date fruit byproducts, diverse fruit peels and their mixtures, some bagasse (coconut bagasse, cassava bagasse, etc.), leaves (banana), pomaces (apple pomace and grape pomace), and cashew apple [119],[172]–[180]. The FFase production was evaluated by *Chrysonilia scatophilia* PSSF84 using banana peel, banana leaf, and other wastes [181]. Authors compared their results with synthetic substrates, and the results corroborate the findings obtained by other researchers [171],[177].

Xylitol (C_5_H_12_O_5_) is a five-carbon sugar alcohol with a sweetness comparable to sucrose but with a 40% lower caloric content. Xylitol is mainly used in the pharmaceutical, cosmetic, dental, and food industries [182]. Banana peels and almond shells are used as a substrate for converting xylose into xylitol (birch sugar) in SSF using *Candida tropicalis* DSM 7524 [47],[120]. It has been reported that mannitol can be produced from celery byproducts [183]. Sorbitol can be produced from apple pomace and apple leaves [184].

### 4.12. Poly (3-hydroxybutyrate), polyhydroxyalkanoate, and biocomposites

Polyhydroxybutyrate (PHB) and polyhydroxyalkanoate (PHA) are biodegradable and biocompatible plastics and an attractive, environmentally friendly alternative to fossil-based thermoplastics like polyethylene and polypropylene [36]. Poly (3-HB) can be produced from orange peel waste as a single carbon source [126]. A PHB concentration of 1.24 g/L culture broth with 41% PHB was acquired in an enhanced medium with a modified *Bacillus subtilis* OK2 strain. Similarly, it was reported that PHA can be produced from papaya waste [185].

Fruit wastes from grapes, apples, olives, bananas, coconut, pineapple, and others have recently been incorporated into polymer matrices to make green composites or films [36],[186]. Various surface treatments of biofilters/fibers could affect the adhesion and applicability of the fillers with different bioplastics. Olive pomace and high-density polyethylene (HDPE) are combined to make green composites. A coupling agent improves interfacial adhesion, allowing the olive pomace to act as a reinforcing material. Recycled HDPE (EHDPE) combines banana fibers (a compatibilizer) and additional filler to generate composites. Banana fibers are non-abrasive, renewable, and cost-effective fillers that can extend the sustainability of an engineering thermoplastic [187]. Likewise, coir fibers and shell particles have been combined with polypropylene to generate composites [188]. In this process, the sustainability of the composites was enhanced, allowing them to be used with biobased and biodegradable polymers. The addition of coupling agents also improved the performance of the fabricated materials.

### 4.13. Bioadsorbents

Bioadsorbents are biological materials that remove heavy metals and other toxic compounds from wastewater and effluents [12]. Several studies have described the absorbent abilities of fruits and vegetable peels (FVPs) and their applications to remove methylene blue dye, malachite green dye, and heavy and other toxic compounds from contaminated aqueous systems and effluents [14],[38]. In this regard, an analysis was carried out using SEM, FTIR, and TGA/DTG to characterize the physicochemical properties of some commonly used FVPs generated from pineapple and pomegranate, among others. Characterization included physicochemical properties and Brunauer–Emmett–Teller (BET) surface area. The BET surface area of FVPs was between 1.0 and 1.4 m^2^/g. Watermelon peel had the highest water absorption potential (11.5 mL/g), while pigeon pea peel had the lowest (5.5 mL/g). All FVPs had acidity of zero charges and a surface pH of 3.0–6.0. The surface of FVPs was irregular and coarse, with pores suitable for immobilization carriers. Thermal analyses revealed that FVPs demonstrate thermal stability below 150 °C. These properties confirm the suitability of FVPs as a low-cost adsorbent [13].

Banana peels were employed as an efficient biosorbent to remove rhodamine B, a water-soluble dye of a fundamental nature [130]. The contact time for rhodamine B adsorption on banana peel powder was 6 min. A bioabsorbent was created using varieties of banana peels in another study to treat palm oil mill effluent and toxic metal ions from polluted water [189],[190]. Similarly, sponge gourd and bottle gourd peel were utilized as adsorbents to eradicate malachite green dye from an aqueous solution and for the adsorption of silver and iron particles, respectively [191]. Watermelon rind is a bio-adsorbent [192]. Date palm empty fruit bunches have been found to have a hexavalent chromium removal of 58.02% at optimum conditions, which depicts its efficacy in bioabsorption [193]. Similarly, grape pomace lignins are processed (pyrolyzed) for heavy metal absorption; it was found that 66.5% Pb could be removed from a substrate with an initial concentration of 300 mg Pb/L [194].

### 4.14. Carbon dots

Fluorescent carbon dots (CDs), which include carbon quantum dots (CQDs) and graphene quantum dots (GQDs), are nanoparticles with a nanometer scale (less than 10 nm but that can be as small as 1 nm) that reflect a new class of carbon materials that can be made using simple synthetic methods [195]. CQDs and GQDs have been utilized in various fields as nanoplatforms for biosensing, bioimaging, optoelectronics, catalytic applications, and drug delivery due to the advantages of these carbon materials [196]. CDs demonstrate improved properties, workability, and environmental sustainability. FVWs have been employed as precursors for preparing ultra-small biomass carbon dots (BCDs) because of their availability, cost-effectiveness, and high quantum yields (QYs). BCDs are made from FVWs in environmentally friendly and straightforward methods that use biomass as a carbon source, such as hydrothermal carbonization and microwave and pyrolysis [14]. The various BCDs prepared from different FVWs and their properties and applications have recently been reviewed [14],[195]. A few representative examples of CD synthesis from FVW and their unique applications are shown in Table 3.

Using a hydrothermal carbonization method at a mild temperature of 180 °C, one-pot fluorescent BCD from orange peel waste was synthesized [132]. The prepared hydrothermal carbons were amorphous. Under UV irradiation, a photocatalyst composed of BCD with zinc oxide was used to degrade naphthol blue-black azo dye, demonstrating superior photocatalytic activity. A low-temperature carbonization process was used to make CDs from mango peels [197]. Analytical techniques such as DLS, UV-visible, FTIR, and fluorescence instruments were used to characterize the synthesized CDs. In a recent study, the multi-color-fabricated CD was obtained from *Manilkara zapota* fruits that display blue, green, and yellow color emissions irradiated with UV light. Due to their ultra-small size and biocompatibility, three CDs act as favorable bioimaging agents for imaging cells. The degree of cytotoxicity of three CDs on HeLa cells shows that they are non-toxic, thereby confirming their safety and biocompatibility. The ultra-small CDs were distributed effectively in the cytoplasm of the cells, suggesting their applications in novel research areas such as cell imaging and biomedical research [14].

A simple, cost-effective hydrothermal process was used to synthesize water-soluble carbon quantum dots (wsCQDs) from lemon peel waste [132]. The synthesized wsCQDs demonstrated good properties, functionality, surface integrity, and high aqueous stability. This wsCQDs-based fluorescent probe could act as an effective and low-cost wastewater treatment material. A new method was devised for synthesizing CD from pineapple wastes, demonstrating its use in sensors, molecular keypad locks, and a memory unit [198]. A new method was developed on photoluminescence immunoassay for the p53 protein using carbon quantum dots encapsulated into silica nanospheres isolated from grapefruit peel [133]. The synthesis, characterization, and bioimaging potential of CDs resulting from three commonly consumed fruits (kiwi, avocado, and pear) were described [195]. To determine the *in vitro* cytotoxicity and potential anticancer effects, human epithelial cells were compared with colorectal adenocarcinoma cells concerning CDs. Additionally, zebrafish embryos were utilized to assess their *in vivo* toxicity, owing to their unique embryonic development, which allows for real-time study as they develop outside of the body. *In vitro* and *in vivo* investigations showed that the synthesized CD displayed toxicity only at high concentrations. The toxicity of kiwi CD was highest in both cell lines and zebrafish embryos, with lower LD50 values. A detailed characterization of fluorescent CD obtained from cauliflower waste peels was performed in another study using different novel techniques. Following this step, CDs were used to detect pesticides, herbicides, and other chemicals. The fluorescence quenching property of CDs was utilized to determine the detection limits of 0.25, 0.5, and 2 ng/mL for diazinon, semicarbazone, and glyphosate, respectively [199].

### 4.15. Bioinsecticides and plant growth regulators

Recently, a few review articles have been published on biopesticides as a green approach to agricultural pest control [200],[201]. These reviews have covered formulation strategies, improvement in action spectra, and modes of action. Rice husk, wheat bran, and other agricultural wastes are used as binding materials for biopesticide formulations; there are very few studies comprising FVWs. We cite here some examples of FVWs as substrates for bioinsecticide production. Bioinsecticides include entomopathogenic fungi such as *Beauveria bassiana*, *Metarhizium anisopliae*, and *Paecilomyces fumosoroseus* [4]. Strains of *B. bassiana* were synthesized in Cuba, using residues from cassava bagasse [202],[203]. The coconut husk residue was deployed for the mass culture of *Bacillus thuringiensis* in SSF [204]. In a recent study, agro-industrial residues (rice husk, apple pomace, whisky draff, soy fiber, rice fiber, wheat straw, beer draff, orange peel, and potato peel) were tested as feasible substrates for fungal conidia production. SSF was conducted at a laboratory scale (100 g) with *B. bassiana* or *Trichoderma harzianum*, whose conidia are reported to have biopesticide properties. The highest productions were 1 × 10^9^ conidia/g dry matter for *B. bassiana* using rice husk or potato peel and higher than 5 × 10^9^ conidia/g dry matter for *T. harzianum* using beer draff, potato peel, or orange pomace [205]. Similar results were obtained in another study using grass clipping and pruning waste for conidia production using *T. harzianum* as the microbial inoculant in SSF [206].

FVWs such as banana and mango peels, coconut husk, and moringa dry leaves are used to produce plant growth regulators [4]. The synthesis of indole 3-acetic acid on cassava bagasse was reported in SSF by *Bacillus subtilis* CM 5, earlier isolated from cow dung microflora [124],[207]. Optimum process parameters for indole 3-acetic acid production (23.5 µg/g dry substrate) were identified as an incubation period of 6 days, pH of 7.0, and moisture-holding capacity of 70% using the response surface methodology technique. Indole 3-acetic acid production was reported using grass clipping and pruning waste using *T. harzianum* in SSF [206].

### 4.16. Antibiotics and antimicrobial agents

Antibiotics are substances formed by various microorganisms that exterminate or selectively impede the growth of other organisms at deficient concentrations [208]. Typical antibiotic industrial development occurs in SmF, but the growing interest in SSF systems has significantly enhanced research into antibiotic production in SSF from FVWs [205],[209],[210].

Penicillin production by *Penicillium chrysogenum* isolated from citrus samples was reported [211]. Lemon, banana, and orange peel waste were deployed to treat infections caused by pneumonia and other Gram-negative bacteria that were multidrug-resistant to antibiotics [205],[209]. Lovastatin production by *Penicillium funiculosum* NCIM 1174 using orange peel in SSF has been reported [123].

### 4.17. Compost/vermicompost and biofertilizers

FVWs are easily converted into compost and vermicompost, making them a low-cost and benign choice for solid waste management. Almost all vegetable roots, tuber crops, and fruit wastes are decomposed into compost and vermicompost by microorganisms and earthworms. Microbial dynamics during the composting of FVW show constant changes during anaerobic digestion [212]. Compost is a strong and natural source of phosphorus, potassium, and nitrogen used to increase and preserve the fertility and composition of cultivation soil [213],[214]. Banana peels are rich in minerals, vitamins, and micronutrients and thus can be used as biofertilizers. Kalemelawa et al. [213] studied the potential of banana peel composting for both waste reduction and nutrient enrichment, particularly in the form of K and N. Research showed that composting banana peels, especially when inoculated with other organic matter like cow dung or poultry litter, leads to faster decomposition under aerobic conditions and results in a compost with high concentrations of K and N. According to a few recent reports, chopped banana peel was co-digested with cow dung for the synthesis of methane-rich gas called biogas by anaerobic digestion [15],[215]–[217]. The high alkaline pH of banana peel compost indicates it may lower soil acidity. Vermicompost, made from banana peels decomposed by earthworm (*Eudrilus eugeniae*), is an eco-friendly, low-cost, and effective biofertilizer that improves plant yield and growth by facilitating nutrient uptake [218]. In a two-year experiment, the vermicompost prepared from cassava and sweet potato bagasse had pretty high levels of essential elements, including nitrogen, potassium, and phosphorus. Sweet potato bagasse-based vermicompost also generated high concentrations of nitrogen, phosphorus, and potassium [219].

### 4.18. Bioethanol and biogas

Horticultural wastes represent a largely untapped resource that could be utilized for biofuel production. Different biofuels can be derived from FVWs, including bioethanol and biogas [220],[221].

#### 4.18.1. Bioethanol

Both yeast and fungi have been widely used as microorganisms for bioethanol production due to their favorable environment in SSF [24]. *S. cerevisiae*, a common yeast, has been extensively reported as a suitable organism for the bioconversion of FVWs into bioethanol under SSF conditions [222]–[226]. Roukas (1994) reported using carob pod as a feedstock for bioethanol production in SSF by *Saccharomyces cerevisiae* [134]. The highest bioethanol concentration of 160 g/kg dry pods and high conversion efficiency were obtained at moderate production conditions. A similar study investigated bioethanol production from mahua (*Madhuca* species) flowers by *S. cerevisiae* via SSF. In another study, Mohanty et al. reported high-quality ethanol, high ethanol production efficiency, and improved fermentation efficiency with mahua flower waste [227]. Apple pomace biomass was employed for ethanol production in SSF using a co-culture of *S. cerevisiae*, *Torula utilis*, and *Candida utilis* [228]. Another study used fruit wastes subjected to SSF for seven days using a co-culture of *A*. *niger* and *S*. *cerevisiae* for ethanol production. According to the findings, pineapple peels had the highest ethanol yield when compared with banana peels, orange peels, and pea pod peels [229]. SSF of banana peels to ethanol was investigated for seven days at different temperatures and pH values using a co-culture of *A. niger* and *S. cerevisiae*. The study confirmed the efficacy of banana peels as the substrate for ethanol production [135]. The feasibility of scaling up the bioconversion of sweet sorghum stalks by *S. cerevisiae* was investigated in a 550 m^3^ rotary drum fermenter, and high ethanol yield and improved product quality were reported [225].

To ensure zero waste generation in a circular bioeconomy scenario, an advanced bioconversion process of potato peel by SSF was proposed for the development of bioethanol and manure [226]. In parts of Asia, palm trunks and banana pseudostems have been successfully utilized to produce high yields of bioethanol through SSF [136],[230]. In contrast to SmF, SSF has been shown to generate higher bioethanol yields. For instance, in the bioconversion of grape and sugar beet pomace, an ethanol yield of 82% was observed after 48 h and was higher than in SmF (72%) [223]. The biorefinery process of conversion of potato wastes to ethanol by SSF was also reported [231]. An interesting study was conducted on exo-microbes to ferment coconut endosperm waste to make it palatable for black soldier fly larvae. Further, it could generate around 40% lipid (w/w), yielding 98% of fatty acid methyl esters of biodiesel upon transesterification [232].

#### 4.18.2. Biogas

The bioconversion of FVW into valuable biogas is a promising solution for waste valorization [41],[46],[215],[233]. The decomposition of FVWs through various microorganisms, such as bacteria and archaea, under anoxic conditions generates biogas as an alternative biofuel [234]–[236].

Carbon-enriched FVWs must co-digest with microbial-rich sources such as cow dung to enhance digestibility and lower retention [217],[237]–[239]. Solid-state anaerobic fermentation of FVWs produces higher biogas yields and eliminates the need for much water in SmF [240],[241]. A mixture of organic waste could be employed to produce high-quality biogas. The actual biogas production starts and advances after a few days of anaerobic digestion (AD) [137].

Papaya peels are suitable for AD [137]. In AD, using papaya peels (after 40–42 days) led to the highest amount of biogas (400 mL) obtained. Meanwhile, 10,473.1 mL of biogas was produced in reactors containing mixtures of wastes in a hydraulic retention time of about 40 days. Biogas synthesized from pineapple waste using cow manure and mixed microbial consortia resulted in a maximum biogas yield with over 70% methane concentration [242].

Thermo-chemical pretreatment is the most effective step for speeding hydrolysis, with the co-digested FVWs producing maximum biogas. In a recent study, it was reported that mixed fruit wastes provided 10% more biogas yield than mixed fruit-vegetable waste. The biogas produced was 63.89% methane, 33.12% CO_2_, and 3% other gases [243]. In a recent study, FVWs were co-digested with slaughterhouse wastewater (as a source of microorganisms/enzymes and nitrogen source) in AD followed by aerobic digestion under optimal conditions for enhancing biogas formation [244]. Therefore, several studies have demonstrated the high suitability of FVWs for biogas production due to their high organic and carbon content.

## 5. Downstream processing and scaling-up aspects

Downstream processing includes all the operational activities following fermentation, such as extraction, purification, recovery, and concentration of the desired product from the solid substrate matrix. Scaling up SSF processes presents unique challenges due to the heterogeneous nature of the solid medium, requiring careful optimization of heat and mass transfer.

Several studies report downstream processing in SSF, leading to upscaling and process development. However, the bulk of these studies mainly focus on enzymes, organic acids, microbial polysaccharides, microbial colorants, bioethanol, and biogas [24],[245]. Since SSF is performed with limited water, extraction with a suitable solvent is necessary to recover the secreted products that bind or are immobilized in the solid matrices. The procedure requires additional facilities representing up to 50% of the product cost [246]. Scaling-up difficulties in SSF are linked to the system's intense heat generation and heterogeneity.

Static processing may require replenishment of evaporated water in certain cases, which can result in an undesired rise in local water activity [27],[246]. Novel information has been produced regarding the effects of process parameters like aeration, which can aid in the clarification of heat and mass transfer impacts. Although the basic design configurations of SSF bioreactors have largely remained unchanged, much new knowledge has been created to change them or other processing aspects for the scale-up process [23]. Future integral valorization of FVWs will most likely be divided into two stages: Direct processing of FVWs into value-added products, followed by processing of the residual streams, byproducts, and leftover matter using traditional waste management technologies such as the production of biogas and bioelectricity as a sustainable strategy for the circular economy [245],[247].

## 6. Life cycle assessment (LCA)

FVWs can serve as a potential substitute for fossil-derived feedstocks for producing platform chemicals, such as biobutanol, bioethanol, hydroxymethyl furfural, and other products (i.e., biogas, biomethane, etc.). However, their environmental performance has not been evaluated adequately. An LCA study was performed to evaluate the environmental burden of citrus waste biorefinery [248]. The functional unit used for LCA was set up as 2500 g of citrus waste processed. The global warming potential was 937.3 kg CO_2_ equivalent per 2500 kg of citrus waste processed.

Recently, the sequential extraction of various products from pomegranate waste with a thorough process examination and an evaluation of the biorefinery's life cycle was analyzed using LCA [249]. The global warming potential of the pomegranate biorefinery is about 4500 kg CO_2_ eq/ton of feedstock processed. The severe hydrolysis step resulted in the emission of toxic gases and significantly contributed to the overall global warming potential. The process reported was successful and a major waste conversion and minimization strategy. The same group studied the impact of LCA on citrus processing industrial waste biorefinery. The functional unit used for LCA was set as 2500 kg of citrus waste processed. The global warming potential was 937.3 kg CO_2_ equivalent per 2500 kg of waste processed. When advanced process intensification technologies such as microwave and ultrasound-assisted steps were utilized to supplant conventional steps, significant decreases in the values of environmental indicators were observed [248]. A material balance for banana peel biorefinery for 1-ton bone-dry banana peel was presented in the LCA framework in an earlier study. The results showed that 432 kg of protein or 170 g of citric acid, 170 kg of pectin, 325 m^3^ of ethanol, and 220 m^3^ of methane could be produced [15].

Various LCA studies confirmed that composting resulted in lower emissions of greenhouse gases and was more environmentally friendly than other biochemical conversion techniques. Successful composting of empty fruit bunches resulted in the generation of products useful for soil amendments and horticultural and vegetable cultivation, thereby contributing to the potential of recycling and bioeconomy approaches. The empty fruit bunch composting with appropriate modifications led to the formation of acceptable compost quality and simultaneously accelerated waste conversion. Finally, a case study on using banana skins as soil amendments to enhance the effective bioconversion of empty fruit bunches and increase nutrient recovery was successfully carried out [250].

Biogas is a product of great interest, being considered a clean energy carrier and a feasible source of bioenergy. Martinez-Ruano et al. studied the techno-economic and LCA of the biogas synthesis process considering Colombian economic conditions [241]. Different process scales were found to have significant potential for biogas processing. Plants with more capacity profit financially more than those with less capacity. However, this advantage comes at the cost of increased energy usage and environmental effects.

## 7. Challenges and perspectives

Bioproducts obtained from FVWs by SSF are an engaging research domain with progressing interests. Most of the reported study outcomes depict the varied uses of FVWs as a substrate for effective valorization. Notwithstanding the progress made so far, it is necessary to expand the frontiers of knowledge and equally explore the various challenges and drawbacks associated with fermentation as a conversion technique. One of the most critical aspects is using appropriate microorganisms in SSF using biotechnological tools and methods such as genetic/metabolic engineering of the native strains to increase productivity, using commensal co-cultures of bacteria and yeasts, and defined microbial consortia. The metabolic landscape of microorganisms available for industrial processes is increasing thanks to biotechnological improvements to enhance their productivity and yield through genetic and metabolic engineering. Fungi, thermotolerant bacteria, and yeasts are being intensively used to produce high-value-added products (Section 4) by using FVW biomass as substrates in SSF. Engineered microorganisms are now capable of using various FVWs as feedstocks, which were previously unable to be processed before their modifications, opening greater possibilities for new markets in places where FVWs are plentifully available. Another important aspect is the development of novel bioreactors, which ensures the reduction of operational constraints and ensures seamless and continuous operation. Despite developments, the primary obstacle to the industrialization of the SSF process is still the absence of effective and readily scalable bioreactors that can effectively handle heat build-up and heterogeneity (mass and heat) while maintaining the highest level of sterility [251]. These challenges arise from the nature of SSF, which involves microbial growth on a solid or partially solid substrate, unlike submerged fermentation. This is probably due to a combination of three factors, i.e., lack of efficient bioreactor design, lack of mathematical models describing the transport and kinetic phenomena at micro- and macroscopic levels, and the lack of effective online process monitoring and control strategies. Although there have been reports in recent years of a small number of bioreactor systems that have partially addressed these issues for a specific application, there still exists a vast scope for improvement to handle a wide range of biotechnological applications [252].

## 8. Conclusions

The benefits and potential of SSF must be assessed for each process/phase. However, some drawbacks to scaling up the process, its microorganisms, substrate/support, parameter selection, and bioreactor designs are predominant. Of course, economic viability depends on carefully comparing SSF and submerged fermentation processes. The prime concerns in the SSF for high-end commercial products include the reproducibility and efficacy of the bioproducts compared to the synthetic products and the cost of the bioproduct to the end-user. Although such bioproducts have been commercialized to some extent, the lack of integration of expertise has been the biggest impediment to commercialization progress. The current time requires an integrated approach to identify the benevolent genomes in novel microorganisms for the overproduction of bioproducts, ensuring reproducibility in upscaling and product efficacy. Apart from the technological aspects, selecting the FVWs for respective bioproducts is vital, and it could be decided based on the biochemical composition of the waste. Hence, the effective valorization of FVWs involves the segregation of FVWs as per the intended bioproduct. Valorization industries could work closely with the local bodies to ensure appropriate sorting. As a novel approach, governments should offer lucrative subsidies to entrepreneurs who want to manufacture novel bioproducts and chemicals from FVWs.

## Use of AI tools declaration

The authors declare they have not used Artificial Intelligence (AI) tools in creating this article.
